# Intraoperative Dexmedetomidine Promotes Postoperative Analgesia and Recovery in Patients after Abdominal Hysterectomy: a Double-Blind, Randomized Clinical Trial

**DOI:** 10.1038/srep21514

**Published:** 2016-02-23

**Authors:** Dong-Jian Ge, Bin Qi, Gang Tang, Jin-Yu Li

**Affiliations:** 1 Department of Anaesthesiology, Huai’an First People’s Hospital, Nanjing Medical University, 6 Beijing Road West, Huai’an, Jiangsu, 223300, P.R. China

## Abstract

Surgery-induced acute postoperative pain and stress response can lead to prolonged convalescence. The present study was designed to investigate the effects of intraoperative dexmedetomidine on postoperative analgesia and recovery following abdominal hysterectomy surgeries. Sixty-four patients scheduled for abdominal hysterectomy under general anesthesia were divided into two groups that were maintained using propofol/remifentanil/dexmedetomidine (PRD) or propofol/remifentanil/saline (PRS). During surgery, patients in the PRD group had a lower bispectral index (BIS) value, which indicated a deeper anesthetic state, and a higher sedation score immediately after extubation than patients in the PRS group. During the first 24 hours post-surgery, PRD patients consumed less morphine with patient-controlled analgesia (PCA) and had lower scores on a visual analogue scale (VAS) than their controls from the PRS group. The global 40-item quality of recovery questionnaire and 9-question fatigue severity score both showed higher recovery scores from day 3 after surgery in the PRD group. with the data are considered together, intraoperative administration of dexmedetomidine appeared to promote the analgesic properties of morphine-based PCA and to expedite recovery following surgery in patients undergoing abdominal hysterectomy.

Postoperative pain and fatigue are two of the key causes of prolonged convalescence following abdominal surgery^1^,^2^,^3^. Opioid based PCA (patient-controlled analgesia) is well established and has been widely used for postoperative analgesia^4^. Currently, the main challenge with PCA is to reduce opioid consumption and the related side effects such as nausea, vomiting, itching, etc. Because surgery-induced fatigue is less understood than post-surgical pain, more investigations are necessary to determine its underlying mechanisms to develop novel drugs or to find effective therapeutics using currently available drugs.

Anesthesia management may modulate surgery-induced pain, stress responses and fatigue^1^,^5^. Recent clinical studies have reported that the highly selective alpha-2 adrenergic receptor (α2-AR) agonist dexmedetomidine promoted an analgesic effect, and prolonged the analgesic time of local anesthetics for up to 24 hours after dental and osteopathic surgeries^3^. Most of these studies investigated the synergic action of intraoperative dexmedetomidine with local anesthetics on surgery-induced acute pain during or following surgeries^3^. However, more evidence is needed to support its potential analgesia-promoting effect in PCA following general-anesthetized surgeries. Few studies have indicated that DEX has an active influence on recovery^6^,^7^, even at a single dose^8^. The evidence noted above suggested that patients with surgery-induced pain and fatigue might benefit from perioperative DEX administration. However, side effects including hypotension and bradycardia have limited its clinical application under conditions without professional monitorings. Therefore, in the present study, we hypothesized that intraoperative DEX would improve the analgesic effect of morphine-based PCA and would promote the recovery following surgery in patients undergoing abdominal hysterectomy.

## Results

### Demographic data and surgery/anesthesia-related information

**Figure 1:**
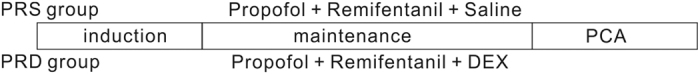
Schematic of anesthesia and post-operative analgesia. Patients received the same treatments for induction and PCA (see the Methods section). Patients in both groups received anesthesia maintenance with propofol, remifentanil and saline (PRS group) or with dexmedetomidine (PRD group).

Patients from both groups had comparable demographic and surgery/anesthesia-related variables, including age, weight, BMI, ASA class, operation time, anesthesia time, and PACU stay time (Table 1). The PRS and PRD patients received either propofol, remifentanil, saline or dexmedetomidine for general anesthesia maintenance and the same treatments for induction and PCA (Fig. 1).

**Figure 2:**
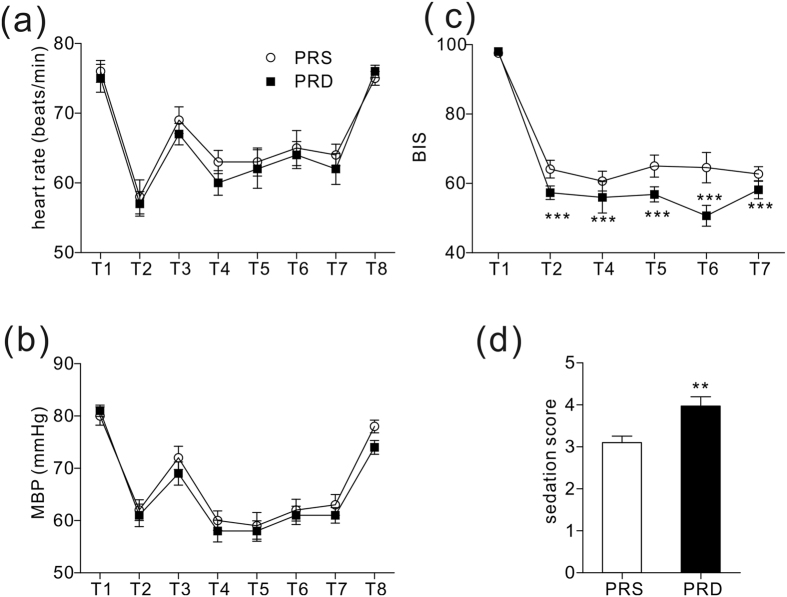
Heart rates, MBP, BIS values and Ramsay sedation scores. (**a**) Heart rates at different time points. (**b**) MBP at different time points. (**c**) BIS values at different time points, ***P < 0.001. (**d**) Ramsay sedation scale score immediately after extubation, **P = 0.004. For Figure 2a–c: T1: baseline, T2: induction, T3: intubation, T4–T7: 10 min, 30 min, 60 min and 90 min after intubation, respectively, T8: 24 hours after surgery.

The two groups were also comparable with respect to their baseline mean blood pressure (MBP) and mean heart rate (HR) before surgery. Furthermore, we observed decreases in MBP and HR induced by induction and sharp increases in MBP and HR evoked by intubation. Subsequently, MBP and HR were maintained at lower levels than baseline to extubation. Moreover, 24 hours after surgery MBP and HR returned to the baseline levels (Fig. 2a,b).

### Anesthesia depth evaluation

Anesthesia depth was monitored with BIS. Significantly, the patients in the PRD group had lower BIS values than those in the PRS group (Fig. 2c, ***P < 0.001), which indicated a deeper anesthesia state. The PRD group also had a higher immediate Ramsay sedation score after extubation than the controls in the PRS group (Fig. 2d, **P = 0.004).

### Postoperative PCA evaluation

**Figure 3:**
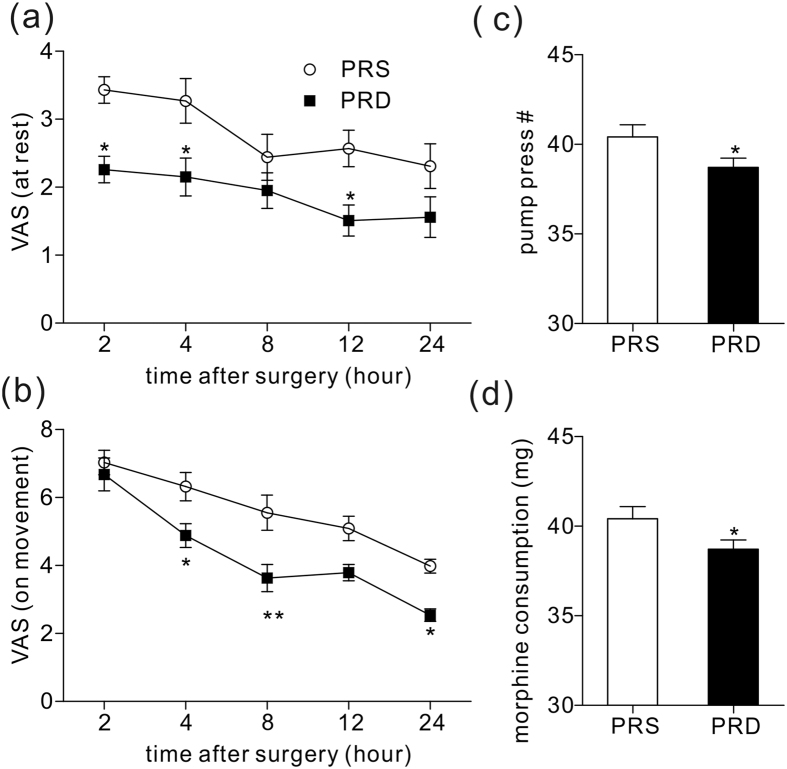
24 hour PCA evaluation and morphine consumption. (**a**) VAS pain score at rest at different time points in the two groups, *P = 0.02, 0.04, 0.03 for time points of 2, 4, 12 hours post-operatively, respectively. (**b**) VAS pain score on movement at different time points in the two groups, *P = 0.03 and 0.02 for time points of 4 and 24 hours post-operatively, respectively, **P = 0.006 for 8 hour post-operative time point. Figure 3c,d show pump press numbers and morphine consumption during the first 24 hours following surgery, *P < 0.05.

After surgery, the patients received a morphine-based PCA pump. Postoperative pain was assessed with a VAS, and the pain-induced pump press number and morphine consumption were noted. During the first 24 hours, patients from the PRD group had a lower VAS score in both the resting (Fig. 3a, P = 0.02, 0.04, 0.03 for time points of 2, 4, 12 hours post-operation time points) and movement state (Fig. 3b, P = 0.03, 0.02 for time points of 4 and 24 hours post-operatively, **P = 0.006 for the 8 hour post operative time point) compared to the PRS group. Patients from the PRS group also had a higher pump press number and more morphine consumption than the PRD group (Fig. 3cd, *P < 0.05).

### Postoperative recovery and fatigue evaluation

**Figure 4:**
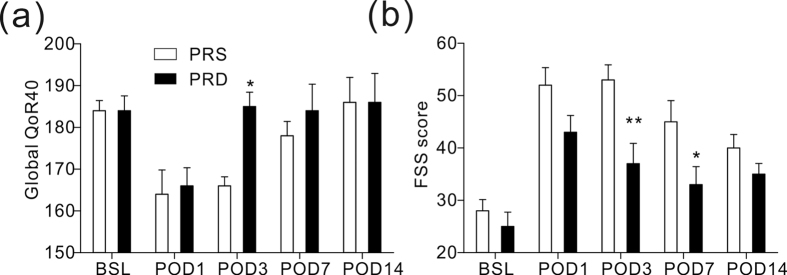
Recovery quality evaluation in the two groups. (**a**) Global 40-item quality of recovery questionnaire score, *P = 0.04 at POD 3 time point. (**b**) Nine-question fatigue severity scores, **P = 0.004 at POD 3 time point, and *P = 0.03 at POD 7 time point. BSL: baseline before surgery, POD: post-operative day.

The global 40-item quality of recovery questionnaire scores showed lower values for the both groups on day 1 after surgery compared with the baseline. On day 3 following surgery, patients in the PRD group had significantly higher scores compared with the PRS group (Fig. 4a, *P = 0.04), but they maintained lower values than their baseline numbers. Patients in the PRD group showed a lower fatigue severity score than those in the PRS group on day 3 (Fig. 4b, **P = 0.004) and day 7 (Fig. 4b, *P = 0.03) after surgery; however, the scores remained higher than their baselines.

### Postoperative adverse effects

No differences were observed in postoperative adverse effects between the two groups during the first 24 hours. The PRD patients trended towards suffering from less adverse effects, such as nausea, vomiting, than those in the PRS group (Table 2).

## Discussion

In the present study, we found that intraoperative administration of dexmedetomidine promoted the analgesic property of morphine-based PCA and the speed of recovery following surgery of patients undergoing abdominal hysterectomy.

It is widely known that approximately 2–10% of patients undergoing abdominal hysterectomy experience severe acute postoperative pain, which can lead to chronic pain^9^,^10^. Opioids, especially morphine-based patient controlled analgesia are widely used for pain control following abdominal hysterectomy^11^,^12^. To combat the side effects, such as nausea, vomiting, itching, etc., there has been a pursuit for novel drugs or for more information regarding combining the currently-available drugs to reduce the morphine consumption. Alpha 2 receptor agonists, like clonidine (α2R:α1R ratio of 200:1), have been used as pain treatments for decades^13^,^14^. A recent study reported that α1 receptor activation encountered α2R-related analgesia and suggested that an agonist with higher α2 R selectivity would show a more potent analgesic effect and would be more suitable for pain treatment^15^. DEX is a α2R agonist developed in the 1990s, and it was first used as a short-term sedative in the intensive care units^5^. Clinical studies have confirmed its potential as an adjuvant for pain treatment, mostly in acute perioperative settings. This use suggests that DEX might be a new drug for surgery-induced acute pain control^11^. In the present study, we combined dexmedetomidine with propofol and remifentanil to maintain the general anaesthesia in patients undergoing abdominal hysterectomy surgery, and we found that intraoperative dexmedetomidine was helpful in relieving both resting and moving postoperative acute pain. Moreover, patients from the PRD group who received intraoperative dexmedetomidine had lower pump-press number, and consumed less morphine than those in the PRS group. The analgesic and opioid-sparing effects of dexmedetomidine have been well described in previous studies both in adults and children^7^,^16^,^17^. Similar to the present data, these studies reported significantly lower VAS scores and morphine consumption and fewer morphine demands. Together with these findings, the present study indicated that intraoperative administration of dexmedetomidine is potentially be used to promote morphine-based PCA following abdominal surgery. Though remifentanil has been reported to induce hyperalgesia following general anesthesia^18^,^19^, we did not see significant difference in consumption of remifentanil and propofol between the two groups (supplementary Figure 1). Thus, we believe that the analgesic effect predominantly came from dexmedetomidine, though not completely.

Dexmedetomidine induces hemodynamic changes, such as hypertension, hypotension, and bradycardia, especially after a loading dose. Thus, in the present study, we administered a continuous infusion without a loading dose. Using this continuous infusion, we did not see significant difference in HR or MBP between the groups. Interestingly, we observed significant lower BIS values in the PRD group during anaesthesia, and higher sedation scores immediately after extubation, which were consistent with previous reports and indicated that intraoperative dexmedetomidine provided more stable anesthesia without changing haemodynamic characteristics, and promoted quality of recovery from surgeries^1^,^6^,^20^.

So far, the mechanisms underlying the long-term analgesic effects of dexmedetomidine have remained unknown. Dexmedetomidine was first introduced into clinical use as a short-term sedative because it is a rapidly-metabolized chemical with a short plasmatic half-time of 2 ~2.5 hours^11^. There are several possible mechanisms underlying the long-term analgesic effect: unlike with the sedation effect, dexmedetomidine uses a different α2AR-dependent downstream mechanism to act as an analgesic. Another reason might be that dexmedetomidine prolongs the analgesic time and analgesic effect of other analgesics. Although an animal study reported that its analgesic properties could be neutralized by the α2AR antagonist^21^, we can’t completely exclude the remote possibility that dexmedetomidine also uses α2AR-independent mechanisms to exert its analgesic effects.

**Figure 5:**
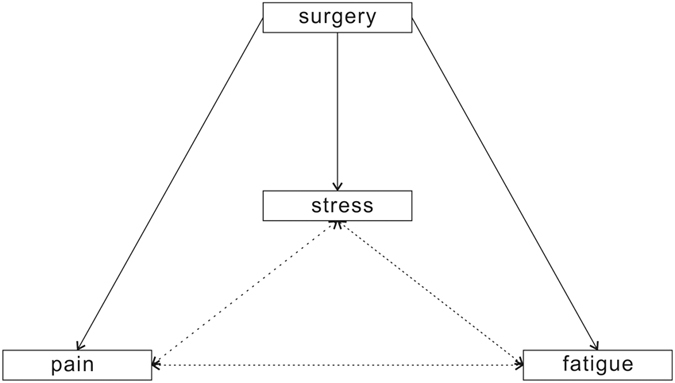
Schematic showing potential relationships among surgery-induced pain, stress and fatigue.

Surgery-induced fatigue was another factor that prolonged convalescence after surgery^1^,^12^,^13^,^14^. In the present study, all of the patients reported higher fatigue level after surgery, but on day 3 and day 7 after surgery, patients in the PRD group had significantly lower scores for fatigue than their controls, consistent with the findings of a recent study from New York University Medical Center^12^. Using a global 40-item questionnaire, the present study found that the Global QoR-40 score was significantly improved in the PRD group on day 3 after surgery. Few other studies have reported that intraoperative infusion of dexmedetomidine had active effects on recovery in patients following different surgeries^12^,^13^,^14^, such as major spinal surgery and nasal surgery. Together with these previous reports, this study indicated that intraoperative dexmedetomidine was helpful in alleviating surgery-induced fatigue in the early postoperative period. Multiple factors are responsible for slow recovery from surgery, including pain, fatigue, and surgery-induced metabolic, endocrine, and immune changes known as ‘stress responses’. There is no existing evidence that shows the relationship among these factors. We believe that there is a vicious cycle among these three factors: acute postoperative pain will reduce movement motivation and keep the patient in a relatively “comfortable position” even for hours, which will cause fatigue to increase and impair the ability to respond to stress physically and mentally. Fatigue might be the result of a multi-system disorder induced by response stress, and it can possibly worsen response stress and acute pain after surgery. Further, at the molecular level, the stress response induces multi-system changes, including inflammatory factors, such as the interleukin cytokines^12^, which are widely accepted mediators of the pain process (Fig. 5). More investigations must be undertaken to verify this hypothesis in the future.

We found that dexmedetomidine induced sedation and analgesia without increasing the risks of opioid-related side effects, such as respiratory depression, consistent with previous reports. We also observed decreasing trends in postoperative nausea and vomiting. Future large sample studies should be performed to verify the effects on morphine- and surgery-related side effects, such as nausea and vomiting.

There might be some limitations of this study: (1) There are four commonly used scales to evaluate sedation level: the Ramsay sedation scale (RSS), the Richmond Agitation Sedation scale (RASS), the Sedation Agitation Scale (SAS), and the Adaption to Intensive Care Environment Scale; they are sufficient for sedation evaluation. We used the RSS, a highly BIS-related scale, to test sedation after surgery in this study. Optimally, future studies should validate the findings of this study using one or more other scales, such as the RASS or SAS, the other two highly BIS-related scales^22^. (2) We used a VAS for post-operative pain evaluation. The numerical rating scale (NRS) is another well-established and widely-used method for pain evaluation, and it was reported to be more reliable than the VAS in some cases^23^. Our hospital is located on the demarcation line between North China and South China, and we received patients from different provinces. The heavy accents with which some patients spoke might have been a limitation to the use of the NRS. For example, some patients from South China often pronounce the number “10” (“Shi” in Chinese mandarin) as “Si”(which is the pronunciation of the number “4”). Furthermore, the anaesthesiologists who performed this study also came from different provinces of the country. Thus, to avoid misunderstanding we used the VAS to evaluate post-operative pain. We nevertheless encourage the NRS to be used in future studies if the conditions are applicable, because it is easier to perform, saves more time and more reliable than the VAS.

Taken together, maintenance with dexmedetomidine (0.4μg/kg/h) provided more stable anesthesia without changing haemodynamic characteristics, and it was useful for promoting morphine-based PCA, alleviating fatigue, and promoting patient recovery following abdominal hysterectomy. The single sex of the patients might be another limitation of this study because the abdominal hysterectomy is a female-only surgery, thus rendering it more difficult to generalize current conclusion to the general population, or at least to male patients. This study indicated that intraoperative administration of dexmedetomidine benefited female patients, at least those experiencing abdominal surgeries.

## Methods

### Subjects

This study was approved by the Institutional Medical Ethics Committee of Nanjing Medical University, and was conducted in accordance with the approved guidelines. Informed consent was obtained from all of the subjects. This study was registered at chictr.org (ChiCTR-TRC-14004313) on February 26, 2014, and was performed at Huai’an First People’s Hospital. The sample size of the study was calculated according to previous studies^24^,^25^, and was based on a pilot study. Twenty-one patients in each group were required to detect a difference of “1 over 10” in the VAS score (primary outcome) with a power of 0.8 and type I error of 0.05^24^. To compensate for dropouts and deviation from normality, 70 patients were enrolled, and assigned to the PRS (n = 29, 6 patients from the PRS group were lost because of noncooperation) and PRD (n = 35) group, using a computer-generated randomized table. The PRS and PRD patients received either propofol, remifentanil, and saline or dexmedetomidine for general anesthesia maintenance. We targeted an 80% probability (β = 0.2) with a significance level of 0.05 and a ~10% dropout rate. The maintenance syringe pumps were prepared by a different anesthesiologist to maintain this study as a randomized, double-blinded investigation. Post-operative evaluations were performed by a different anesthesiologist. Patients matching the following criteria were included in this study: between 35 and 65 years old; an American Society of Anesthesiologists (ASA) grade I or II; weight 45–75 kg; and height 145–170 cm. Patients were excluded if they had ischaemic heart disease; opioid addiction, long-term alcohol abuse, long term smoking history, sedative–hypnotic drug(s) use; obesity (BMI > 30); a history of postoperative nausea and vomiting; neuropsychiatric diseases or a related treatment history. Patients were instructed in the use of the visual analogue scale (VAS; 0, no pain, to 10, worst possible pain) and the *i.v.* PCA pump (50 mg morphine and 8 mg ondansetron in 100 ml saline, every pump press resulting in a 2 ml infusion). No important changes to the methods were made after trial commencement. Full details of the trial protocol can be found in the supplementary appendix.

### Anesthesia

On arrival, electrocardiography, blood pressure, oxygen saturation, and the bispectral index (BIS) were monitored every 5 minutes. A BIS value < 60 was used to adjust the titration of anesthetics on the basis of amnesia. For induction, patients from the both groups received midazolam (0.05 mg/kg), remifentanil (2–5 μg/kg), propofol (1.5–2 mg/kg), and cisatracurium (0.2 mg/kg). Immediately after intubation, the patients were ventilated with an oxygen and air mixture (FiO_2_ = 0.4) with a PetCO_2_ of 30–35 mmHg. Intravenous infusion was switched to a maintenance syringe pump at rate of 50–80 μg/kg/min for propofol, 0.15–0.2 μg/kg/min for remifentanil, and 0.4 μg/kg/h for dexmedetomidine. Cisatracurium (0.05 mg/kg) was intermittently used for muscle relaxation. The patients were awakened and extubated followed by sedation evaluation using the Ramsay sedation scale.

### Data collection

Patient demographic information was collected on admission. Haemodynamic indices and BIS were recorded during surgery every 5 min, and data from selected time points were used for analysis. Postoperative pain at rest and during movement were evaluated with a VAS, and the global 40-item quality of recovery questionnaire^26^,^27^ and the 9-question fatigue severity score^1^ were used to evaluate the recovery and fatigue level at different time points post-surgery (for all time points see figure legends). Subjects who received rescue morphine in the PACU had the rescue morphine included in the total consumption of postoperative PCA morphine. PCA pump pressing numbers and adverse effects after surgery were noted.

### Statistics

All of the data in the present study were analyzed with GraphPad Prism software, version 5.0. Parameters such as age, weight, operation time, anesthesia time and PACU stay time, pump-press number and morphine consumption were compared between the two groups using unpaired *t* test. HR, MBP, VAS, and BIS at different time points were compared between the two groups by *two-way ANOVA,* followed by *Bonferroni’s post-test*. ASA grade and postoperative adverse effects were analyzed with *Fisher’s test*. All data with P < 0.05 were considered statistically significant.

## Additional Information

**How to cite this article**: Ge, D.-J. *et al.* Intraoperative Dexmedetomidine Promotes Postoperative Analgesia and Recovery in Patients after Abdominal Hysterectomy: a Double-Blind, Randomized Clinical Trial. *Sci. Rep.* **6**, 21514; doi: 10.1038/srep21514 (2016).

## Supplementary Material

supplementary figure 1

## Figures and Tables

**Table 1:** Basic demographic data and surgery/anesthesia-related information.

	PRS group	PRD group	P value
Age (years)	52.6 ± 2.2	53.0 ± 1.9	0.90
Weight (kg)	60.6 ± 1.8	61.1 ± 1.9	0.87
BMI (kg/m2)	22.9 ± 0.7	23.6 ± 0.6	0.50
ASA I/II	19/10	22/13	1.00
Operation time (min)	117.8 ± 5.8	115.3 ± 6.7	0.78
Anesthesia time (min)	155.1 ± 6.4	157.3 ± 8.0	0.83
PACU stay time (min)	33.2 ± 2.6	26.4 ± 3.0	0.45

Data are shown as the mean ± s.e.m.

**Table 2:** Postoperative side effects of the patients in the two groups.

	PRS group	PRD group	P values
Nausea	11/18 (37.93%)	7/28 (20.00%)	0.16
Vomiting	8/21 (27.59%)	5/30 (12.50%)	0.13
Itching	2/27 (6.90%)	3/32 (8.57%)	1.00
Respiratory depression	0/29 (0.00%)	0/35 (0.00%)	–
Dizziness	4/25 (13.79%)	4/31 (11.43%)	1.00
Bradycardia	3/26 (10.34%)	3/32 (8.57%)	1.00

Data shows the positive numbers and percentage of patients.
