# Supplementary material for: Intraoperative Dexmedetomidine Promotes Postoperative Analgesia and Recovery in Patients after Abdominal Hysterectomy: a Double-Blind, Randomized Clinical Trial
Source: Sci Rep. 2016 Feb 23;6:21514. doi: 10.1038/srep21514 (PMC4763240; doi:10.1038/srep21514)
Supplement: supplementary figure 1 [file srep21514-s1.pdf]

supplementary figure 1

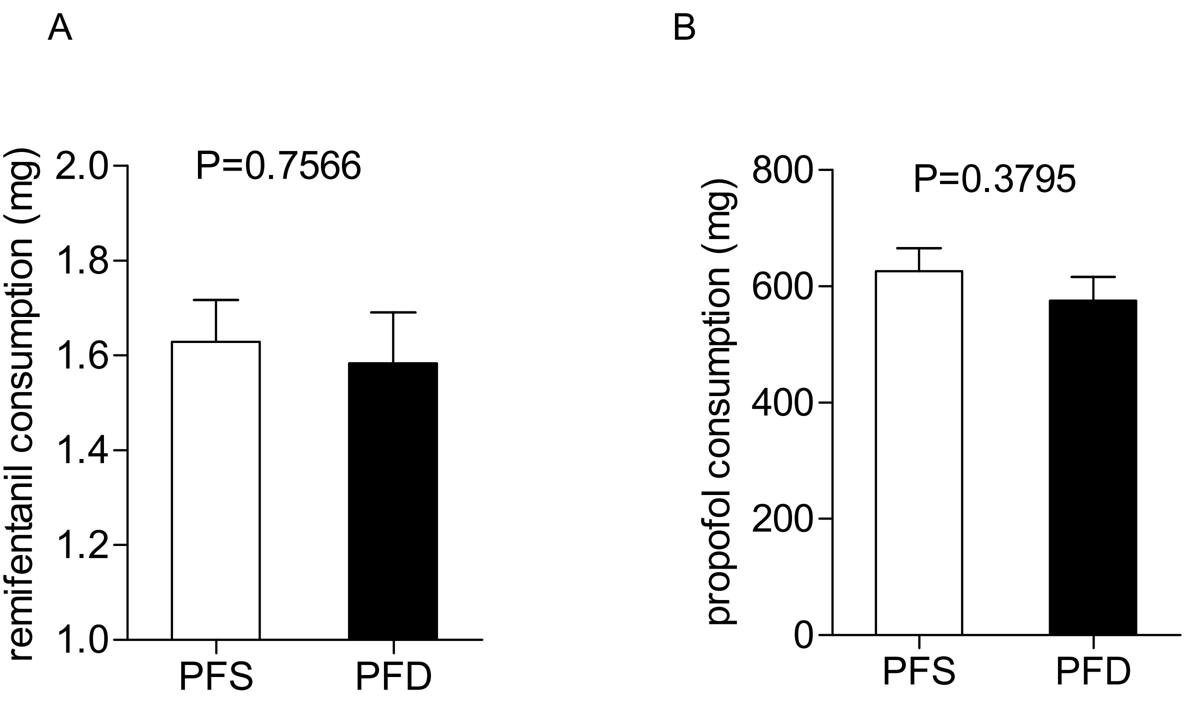

legend:
A: total consumption of remifentanil.
B: total consumption of propofol.
